# Did Media Attention of the 2009 A(H1N1) Influenza Epidemic Increase Outpatient Antibiotic Use in France?: A Time-Series Analysis

**DOI:** 10.1371/journal.pone.0069075

**Published:** 2013-07-24

**Authors:** Adeline Bernier, Caroline Ligier, Didier Guillemot, Laurence Watier

**Affiliations:** 1 INSERM, U 657, Paris, France; 2 Institut Pasteur, PhEMI, Paris, France; 3 Université de Versailles Saint Quentin, Faculté de Médecine, Paris Ile de France Ouest, France; 4 AP-HP, Hopital Raymond Poincaré, Unité Fonctionnelle de Santé Publique, Garches, France; National Institutes of Health, United States of America

## Abstract

**Background:**

In France, the 2009 A(H1N1) influenza epidemic occurred between September 2009 and January 2010. Sparking widespread controversy, it was intensely reported in the media. Despite therapeutic inefficacy, antibiotic consumption and viral respiratory infections are positively correlated, particularly in France, where antibiotic overconsumption is well-known. We first determined the period when media coverage was high, and then compared, during this period, observed outpatient antibiotic consumption to estimated outpatient antibiotic consumption “without media attention”.

**Materials and Methods:**

To evaluate media coverage, two online databases were consulted: Factiva and Europresse. To quantify outpatient antibiotic consumption, we used data on reimbursements of outpatient systemic antibiotics from the computerized databases of the two main National Health Insurance agencies. Influenza-like syndromes data came from the French GPs Sentinelles Network. Weekly time-series of antibiotic consumption were modeled by autoregressive moving-average models with exogenous inputs and interventions. Analyses were computed for the entire series and by age group (0–5, 6–15, 16–60, and >60 years).

**Results:**

Media coverage was intense between April 2009 and January 2010. No effect on total outpatient antibiotic consumption was observed during the whole mediatic period. However, during the epidemic in France (September 2009-January 2010), we found an antibiotic underconsumption for the entire series, 0–5 and >60 years. Additionally, at the beginning of the pandemic, when cases were still outside France (June 2009-August 2009), we found an antibiotic overconsumption for patients >16 years.

**Conclusion:**

The early period of A(H1N1) virus circulation compared with seasonal influenza or an overdeclaration of ILS cases might explain the antibiotic underconsumption observed during the period of active A(H1N1) virus transmission in France. At the pandemic onset, when uncertainty was high, the overconsumption observed for individuals >16 years might have been caused by alarmist media reporting. Additional analyses are needed to understand the determinants of antibiotic consumption during this period.

## Introduction

During the last few decades, the emergence and dissemination of multidrug-resistant bacterial strains have become a major public health issue worldwide [1], [2]. For years, doctors have been prescribing (and patients have been demanding) antibiotics for viral respiratory infections, eg colds and influenza, even though antibiotics do not cure viral infections [3], [4]. This overuse of antibiotics has been the main driving force in the spread of multiresistant bacteria. In the community, β-lactam–resistant *Streptococcus pneumoniae* is one of the best-known bacteria that has spread during the past 20 years, and, more recently, *Enterobacteria* producing extended-spectrum β-lactamase has become a major worry [1]. Indeed, in the 2000s, the highest European rate of *S. pneumoniae* antibiotic resistance was observed in France, which also has one of the highest rates of community antibiotic consumption, more than three-fold higher than the Netherlands [5], [6]. To fight against this overuse, the French government launched, in 2001, a national program “Keep antibiotics working”. Since 2002, a public service campaign (“Antibiotics are not automatic!” and “Antibiotics, if used wrongly, loose their potency!”) is run every winter from October to March. In 2007, an evaluation of this campaign showed that winter outpatient antibiotic use decreased by 26·5% compared to that of the 2000–2002 pre-campaign period [7].

In April 2009, the A(H1N1) virus emerged in the USA and Mexico. It spread throughout the world very quickly, resulting in outbreaks in more than 200 countries and claimed the lives of more than 18,000 persons [8]. According to the French Institute for Public Health Surveillance (InVS), 1,334 severe cases and 312 deaths were reported in France, and, in terms of number of symptomatic cases and their severity, this pandemic influenza was not worse than annual influenza epidemics [9]. Because of this virus's atypical features and the highly controversial management of this pandemic by the French government, media coverage was omnipresent during several months. Because antibiotics can be useful to prevent influenza bacterial complications, the indirect impact of this media coverage on antibiotic prescriptions warrants examination. Herein, we report the results of an assessment of the media coverage during the A(H1N1) pandemic influenza period in France, as well as a comparison between the observed outpatient antibiotic use with media attention and a model-based estimated outpatient antibiotic use “without media attention”.

## Methods

### Data sources

The French National Health Insurance (NHI) program covers all medical care provided by outpatient and private practice physicians and pharmacies. We obtained aggregated 2000–2010 data on all antibiotics prescribed, dispensed by outpatient and private pharmacies, and reimbursed computed from anonymous computerized individual files in the databases of the two main NHI agencies that cover salaried workers and the self-employed (>85% of the French population). Each file contains drug-related information, prescription date, patient’s sex, year of birth and region of residence. The study concerned only systemic antibiotics (anatomical therapeutic chemical class J01) used in the community. Demographic data were obtained from the French National Institute for Statistics and Economic Studies (INSEE, http://www.insee.fr). The results are presented as weekly rates of antibiotic prescriptions per 1,000 inhabitants.

Weekly influenza-like syndrome (ILS) incidence was provided by the French Sentinelles Network (http://websenti.u707.jussieu.fr/sentiweb) [10]. This network defines ILS as the combination of the following clinical symptoms: sudden onset of fever ≥39°C, myalgias and respiratory symptoms, eg dyspnea and/or cough. The data are presented as ILS incidence per 100,000 inhabitants.

This work considered aggregated data and not individual ones. Thus, no ethic statement or patient consent was involved.

Two online databases were used to assess media coverage of the A(H1N1) pandemic: Factiva (http://www.dowjones.com/factiva/index.asp) and Europresse (http://www.europresse.com). These online databases provide access to newspaper articles of many French and international newspapers. We conducted a search on articles published in two major national daily newspapers, *Le Monde* and *Le Figaro*, between January 2009 and August 2010, in order to retrieve and count the number of A(H1N1) influenza-related articles published in these two newspapers. Results are presented as the number of articles published in these two newspapers per week.

### Statistical analyses

The media period began when at least 5 articles per week were released and ended when fewer than 5 articles were published over 3 consecutive weeks. This threshold appeared suitable considering the number of newspapers considered. The virus emerged in April 2009, so the media period started, at the earliest, in April.

We conducted time-series analyses to predict what antibiotic consumption during the A(H1N1) influenza would have been without media coverage [11], [12]. To take into account ILS incidence and public health campaign effects on antibiotic consumption, an autoregressive moving-average model with exogenous variable (ARMAX) including intervention functions was used [13], [14].

The intervention ARMAX models were built in two steps. The first concerned the 2000–2002 period, before the first public health campaign, considering antibiotic consumption during it as the baseline. Because of seasonal fluctuations, a trigonometric function had to be estimated and removed to render the residual series in a stationary mode. We then fitted an autoregressive moving-average (ARMA) model to the observed data. The second step evaluated the impact of the public health campaigns on antibiotic consumption between October 2002 and March 2009. As those campaigns were held every year at the same period of the year since 2002, we assumed that they did not modify seasonal fluctuations or change the ARMA model structure, but affected only the means. We added 13 dummy variables (seven for each annual campaign (c_1_ to c_7_) and six for the rest of the time (r_1_ to r_6_)) to the ARMA model identified in step 1. The ILS incidence was added to that model using a simple transfer function (linear function). This allows estimating an average effect of the ILS incidence during the entire period. To select the models, we first tested the independence of the residual series (Ljung & Box test) and its Gaussian distribution (Shapiro-Wilk test). When two or more models were identified, we used Schwartz information criterion and choose the one which minimize that criterion. Every step of model identification is detailed in §1 and 2 in file S1.

**Figure 1:**
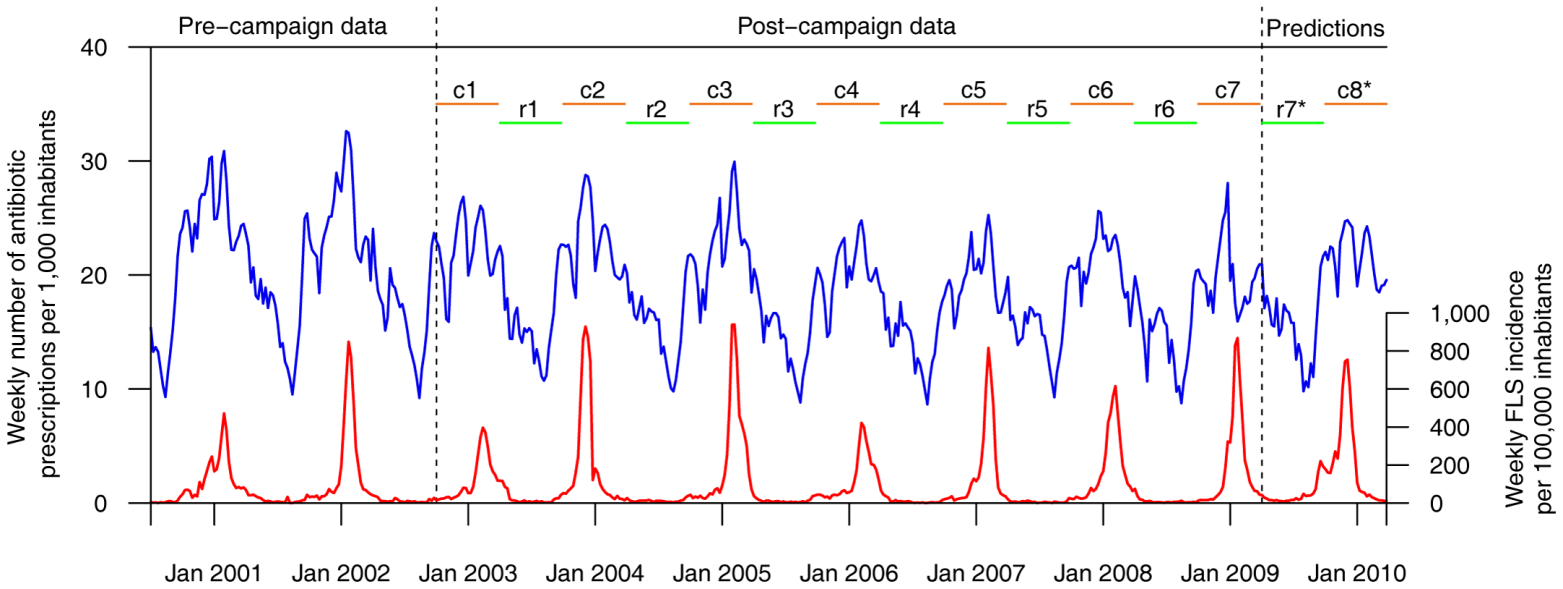
Weekly number of antibiotic consumptions, periods and intervention functions used to construct the ARMAX model. *Parameters estimated using the tendency since 2002 or the last observed one if it had changed. Dashed vertical lines separate the data periods and model predictions.

To predict antibiotic consumption, the impact of the campaigns during A(H1N1) influenza media coverage had to be known (r_7_, c_8_) (figure 1). We then defined two “extreme” scenarios to describe those impacts, using a “stationary” hypothesis (we considered the impact of the campaigns in 2009–2010 would be the same as the one estimated in 2008–2009) and an “evolutionary” hypothesis (we predicted an impact of the campaigns in 2009–2010 taking into account the impact the campaigns had since 2002) (see §3 in file S1). Expected antibiotic consumption “without A(H1N1) influenza media coverage” was predicted for each scenario. Three numbers were calculated: an “expected number” corresponding to the sum of predicted weekly antibiotic consumptions for each scenario during the study period, and an “observed number” corresponding to the sum of observed weekly antibiotic consumptions. For expected numbers, 95% confidence intervals (CI) were calculated using the sum of variances of the forecasts during the study period. A “final” CI was obtained using the ranges of the two 95% CI (see §3 in file S1). Only observed numbers and final CI are presented here.

Analyses of the entire series and by age group (0–5, 6–15, 16–60, and >60 years) were computed with SAS^®^ 9.1, R2.13.1 and Excel^®^ 2010 softwares.

## Results

**Figure 2:**
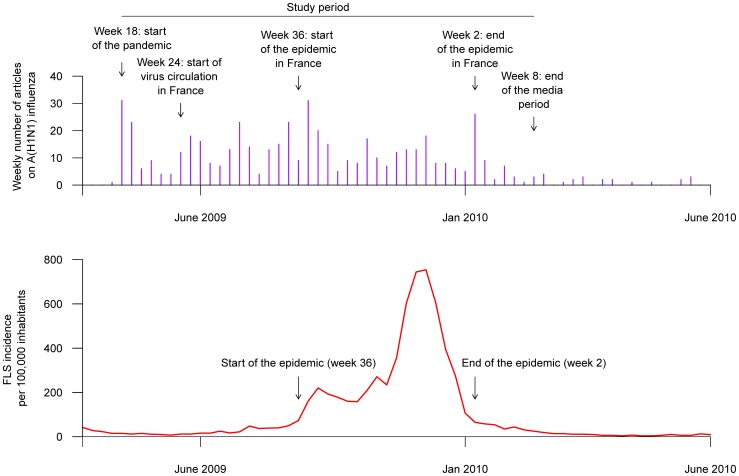
Weekly number of articles published on A(H1N1) and ILS incidence*: January 2009 – August 2010. *influenza-like syndrome incidence per 100,000 inhabitants.

**Figure 3:**
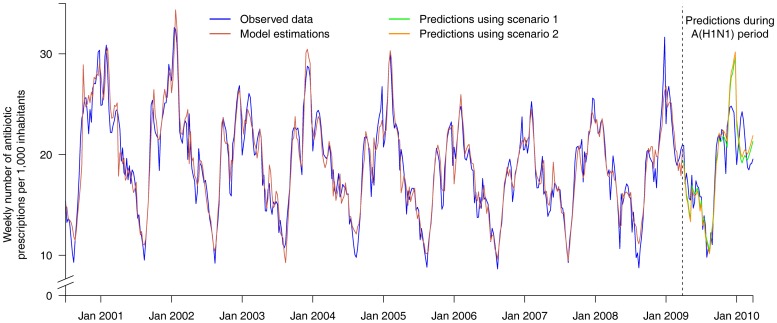
Weekly number of antibiotic prescriptions*: fit of the model and predictions for both scenarios^#^. *per 1,000 inhabitants. ^#^Scenario 1 corresponds to the “stationary” hypothesis and scenario 2 to the “evolutionary” one.

**Figure 4:**
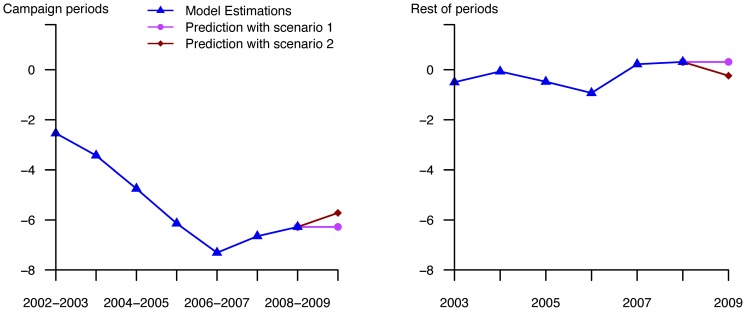
Estimated and predicted changes for both scenarios^#^ of intervention functions for the entire series. ^#^ Scenario 1 corresponds to the “stationary” hypothesis and scenario 2 to the “evolutionary” one.

A total of 530 articles on the A(H1N1) virus were released between January 2009 and August 2010. According to our criteria, media coverage was intense during the 42 weeks beginning in April 2009 (week 18) and ending in February 2010 (week 8) (figure 2), with 508 articles published. A study of the number of weekly articles published in the same two newspapers on seasonal influenza since 2000 showed that this number never exceeded 5 (data not shown). Between April and early June 2009 (weeks 18 to 23), when no A(H1N1) virus was circulating in France, 77 articles were released. During the period of low virus circulation in France (number of cases below the epidemic threshold), ie June–August 2009 (weeks 24–35), 166 articles were released. During the A(H1N1) epidemic in France (September 2009–January 2010 (weeks 36–2)), 240 articles were published. Considering antibiotic consumption, an intervention ARMAX model fulfilling goodness of fit criteria was obtained for each series (see figure 3 for entire series and figure S1 for other considered series). All the models are detailed in file S1. Scenario estimations are described in figure 4 for the entire series (and figure S2 for the other considered series). Using scenario estimations, antibiotic consumption “without A(H1N1) influenza media coverage” was finally predicted (figure 3 and figure S1). The observed numbers and final CI covering the whole period of A(H1N1)-influenza circulation, the period when no cases were reported in France, and the epidemic period in France are given in table 1. For the whole period, no differences between observed and predicted numbers were found for the five series examined. However, when we studied more specific periods, we did notice differences between these two numbers. During the epidemic period in France (weeks 36–2), we observed an underconsumption (ie the observed number was lower than the predicted one) for entire series, subjects 0–5 and >60 years old. Underconsumption varied from 2% to 10% for entire series, from 6% to 14% for the youngers and from 2% to 14% for the olders. Additionally, during the period when there were no cases in France (weeks 18–23), we observed an overconsumption (ie the observed number was higher than the predicted one) for individuals >16 years, with an increase that varies from 1% to 20%.

**Table 1:** Antibiotic consumption in France during the three periods of A(H1N1)-influenza virus activity in France, according to age group.

Age-group	Period		
	Whole A(H1N1) circulation	Without cases in France	Epidemic in France
Entire series	794·6 [774·5–823·5]	113·6 [98·2–118·3]	407·5 [414·2–451·4]*
0–5 years	1656·4 [1530·4–1745·3]	209·8 [158·3–243·2]	921·6 [976·7–1068·8]*
6–15 years	696·6 [658·0–737·8]	91·3 [67·7–99·9]	368·9 [356·8–403·4]
16–60 years	718·5 [684·4–740·1]	103·7 [86·9–102·6]**	365·9 [356·6–393·3]
>60 years	763·2 [750·5–829·3]	120·3 [100·5–119·0]**	366·7 [375·7–424·6]*

Values are observed numbers (sum of weekly antibiotic prescriptions per 1,000 inhabitants) [final CI corresponding to the ranges of the two 95% CI of the sums of forecasts during the study period].

* Observed value lower than the final CI.

** Observed value upper than the final CI.

## Discussion

The link between viral infection and antibiotic consumption has now been very well-documented in numerous studies [3], [4]. This association was explored for seasonal influenza but never for a pandemic influenza, with exceptionally high media coverage and for which health effects were still uncertain. In this context, antibiotic-consumption–related behavior was totally unknown and not predictable.

The data used in this study were provided by two different sources. Antibiotic-prescription reimbursements came from NHI agencies, assuring reliable and thorough data recording during the pandemic. ILS-incidence data was provided by the Sentinelles Network. This network estimates ILS incidence relying on declaration of general practitioners, without virological confirmation. During the A(H1N1) pandemic, they declared that the epidemic threshold was crossed week 36–2009. Another surveillance network (GROG), relying on samples to estimate virologically confirmed influenza number of cases, declared the start of the influenza epidemic in France week 47–2009. This discrepancy suggests there might have been an overdeclaration of ILS by general practitioners in September and October 2009, leading to an overestimation of ILS incidence by Sentinelles Network. Some studies suggest that the increase in ILS in September and October 2009 was caused by respiratory viruses other than influenza (eg rhinovirus) [15]–[18].

To determine the period of A(H1N1)-influenza–related media coverage in France, we only examined articles from two national newspapers available in two online databases, but no local newspaper coverage, thereby probably underestimating the overall coverage of this period. However, first, because our objective was not to quantify media coverage but to determine the trend and identify the period of most intense media coverage, we considered that examination of only two national daily newspapers was sufficient; second, the comparison with weekly television-news time (http://inatheque.ina.fr) devoted to A(H1N1) indicated the same media period (data not shown).

Intervention ARMAX models are well-adapted to time-series analyses. We could construct weekly time-series covering more than 10 years. For each series, models were identified independently and similar ARMA structures were found, supporting confidence in their validity. The main limitation of this study concerns the predictions of the two parameters of the intervention functions after April 2009 (ie the prediction of the impact of the campaigns in 2009–2010). The observed impact of the campaigns between 2002 and 2009 were very different depending on the series; however, another analysis of time series until June 2010 yielded estimated values in accordance with our predicted ones (unpublished personal data). For our predictions, we tried to adhere closely to the observed tendency, and used two scenarios to estimate forecasts and their 95% CI limits to avoid falsely significant results.

Media attention was strong for almost 10 months (42 weeks). During that long period, no change in overall antibiotic consumption was observed, which might reflect a loss of power because prediction 95% CI, and thus the final used, widened with the time. When considering a shorter media-coverage period (18 weeks), corresponding to high A(H1N1)-virus circulation in France, underconsumption was estimated for the global antibiotic consumption, and for subjects 0–5 and >60 years old. This observed underconsumption reached −14%. Considering the antibiotic-consumption profile during 2009–2010, no antibiotic-prescription peak was seen when the ILS incidence was at its maximum, which had not been observed in any previous years. Several hypotheses can be made to explain this model-estimated result. First, as previously mentioned, the ILS incidence might have been overestimated; then adjusting to it led to an overestimation of expected antibiotic use, and consequently an observed “underconsumption” as the predicted number was higher than the observed one. Second, due to the early arrival of the pandemic influenza virus in France (September 2009) compared to seasonal influenza (December–January), general practitioners’ offices might have been less saturated than usual during the A(H1N1) period of activity as classical winter viruses were not circulating yet, generating more careful antibiotic prescription [19], [20]. This hypothesis would explain the shape of the outpatient antibiotic consumption curve during this 2009–2010 winter, with three “medium” peaks instead of one “big” peak and two small ones.

Finally, over the 6-week period without A(H1N1) virus circulating in France, overconsumption which varies from 1% to 20%, was observed for individuals >16 years. We here only assessed what would have been the antibiotic consumption “without media attention” and thererefore cannot draw any formal conclusion on the link between media attention and the observed overconsumption for individuals >16 years during this period. However, subjects 16–60 years old might have been affected by the omnipresent media coverage, because unlike seasonal influenza, it corresponds to the age category for which deaths of people without any risk factors were seen. It has already been previously documented that media coverage can influence medical care and drug consumption [21], [22]. Consequently, one hypothesis to explain this observed overconsumption during this period would be the alarmist media attention related to the circulation of the A(H1N1) virus worldwide. For those >60 years old, because antibiotic consumption has increased since 2007 outside campaign periods, healthcare services might have been perturbed during this period with excessive antibiotic prescriptions, even in the absence of respiratory viruses. Media attention might be one factor having influenced antibiotic consumption in this age group during this period. Additional specific studies are needed to understand the determinants of antibiotic consumption of individuals >16 years during this period.

In conclusion, despite media attention and uncertainty about health effects, no upsurge of antibiotic prescriptions was observed for the whole study period, and in particular when the pandemic virus circulated in France. However, early during the pandemic, when the uncertainty was high, increased antibiotic use for individuals >16 years was observed. Alarmist media reporting might have heightened the worries of those individuals, resulting in increased antibiotic use.

## Supporting Information

Figure S1 Weekly number of antibiotics prescriptions*: fit of the model and predictions for both scenarios^#^ for age-group series. *per 1,000 inhabitants. ^#^Scenario 1 corresponds to the “stationary” hypothesis and scenario 2 to the “evolutionary” one.
(TIFF)

Figure S2 **Estimated and predicted changes for both scenarios^#^ of intervention functions for age-group series.** ^#^ Scenario 1 corresponds to the “stationary” hypothesis and scenario 2 to the “evolutionary” one.
(TIFF)

Text S1 **Identification of intervention ARMAX models.**
(PDF)
