# Supplementary material for: Did Media Attention of the 2009 A(H1N1) Influenza Epidemic Increase Outpatient Antibiotic Use in France?: A Time-Series Analysis
Source: PLoS One. 2013 Jul 24;8(7):e69075. doi: 10.1371/journal.pone.0069075 (PMC3722179; doi:10.1371/journal.pone.0069075)
Supplement: Text S1 — Identification of intervention ARMAX models. (PDF) [file pone.0069075.s003.pdf]

Supporting text S1

Adeline Bernier[1,2,3], Caroline Ligier[1,2,3], Didier Guillemot[1,2,3,4], Laurence Watier[1,2,3*]

[1]Inserm, U657, Paris, France,
[2]Institut Pasteur, PhEMI, Paris, France,
[3]Université Versailles Saint Quentin, Faculté de Médecine Paris Ile de France Ouest, EA 4499, France,
[4]AP-HP, Hôpital Raymond Poincaré, Unité Fonctionnelle de Santé Publique, Garches, France.

Identification of intervention ARMAX models:

An ARMAX model was retained for each series. The different steps of model identification are detailed.

When using intervention ARMA model, the first step is the identification and estimation of a model before the intervention. Moreover, to identify an ARMA model, time series has to be in a stationary mode. Since antibiotics consumption presents a seasonality with a peak in winter, a first step consists in removing that non-stationary phenomenon to the observed series.

1 Pre-intervention period: June 2000 to October 2002

Let X_t the considered weekly time series for $t = 1$ to 118.

1.1 Stationarity

Centering

Let μ_w corresponding to "winter" mean of X_t, estimated between week 40 of year n and week 13 of year $n+1$ and μ_s corresponding to the "summer" mean of X_t, estimated between week 14 and week 39 of year n. The total mean is defined by:

$$\mu = \mu_w \prod_{i=1}^{2} w_i + \mu_s \prod_{i=1}^{3} s_i$$

where
$w_1 = 1$ if week 40 of year 2000 $\leq$ t $\leq$ week 13 of year 2001; 0 otherwise,
$w_2 = 1$ if week 40 of year 2001 $\leq$ t $\leq$ week 13 of year 2002; 0 otherwise,
$s_1 = 1$ if t $\leq$ week 39 of year 2000; 0 otherwise,
$s_2 = 1$ if week 14 of year 2001 $\leq$ t $\leq$ week 39 of year 2001; 0 otherwise,
$s_1 = 3$ if week 14 of year 2002 $\leq$ t $\leq$ week 39 of year 2002; 0 otherwise.

Seasonal component

From the centered series $(X_t - \mu)$, a trigonometric function of period 26 and 52 was estimated:

$$f(t) = \alpha_{c52} \times \cos\frac{2\pi t}{52} + \alpha_{s52} \times \sin\frac{2\pi t}{52} + \alpha_{c26} \times \cos\frac{2\pi t}{26} + \alpha_{s26} \times \sin\frac{2\pi t}{26}$$

Only significant parameters were kept.

1.2 ARMA model identification

From the stationary time series, $Y_t = X_t - \mu - f(t)$, the model was identified with Box & Jenkins algorithm. Using the "Auto-Correlation Function" (ACF) and the "Partial Auto-Correlation Function" (PACF), we

included, step by step, autoregressive or moving average parameters, beginning with those corresponding to short-term dependencies (between 1 and 26 lags). Non significant parameters were removed from the model. New parameters were included until residuals, ϵ_t, were Gaussian white noise (not correlated Gaussian residual series, with zero mean and constant variance). Correlations were testing using Ljung & Box test which allow to test all first K estimated values of the ACF; K=102 were considered, close to two seasonal periods, as suggest by Box & Jenkins. Shapiro-Wilk test was used for testing Gaussian distribution, since it is sensitive to tails of the distribution, it was sometimes necessary to remove a few "extreme" residuals, in order to not reject Gaussian distribution. When two or more models were identified, we used the one which minimized Schwartz information criterion.
A general writing of the ARMA model could be:

$$Y_t = \Theta(B)(\Phi(B))^{-1}\epsilon_t \qquad \epsilon_t \sim N(0, \sigma_\epsilon^2)$$

where
$Y_t = X_t - \mu - f(t)$,
B is the backshift operator, $B^k X_t = X_{t-k}$,
$\Phi(B) = (1 - \phi_1 B^1 - \phi_2 B^2 - \ldots - \phi_p B^p)$ related to AR(p) process,
$\Theta(B) = (1 - \theta_1 B^1 - \theta_2 B^2 - \ldots - \theta_q B^q)$ related to MA(q) process,
ϵ_t the residual series.

Not to overload the writing, one factor for each process was supposed. However, multiplicative factors could be identified. Unlike a model involving a single additive factor, the introduction of multiplicative factors can be modeled as a large number of dependencies between observations without estimating all parameters. This is consistent with the parsimony principle advocated by Box & Jenkins.

1.3 In brief

For each considered times series, a global mean μ, a trigonometric function $f(t)$ including only significant parameters, as well as a specific ARMA model were estimated.

2 Entire period: June 2000 to March 2009

Let X_t the considered weekly time series for $t = 1$ to 455.

2.1 Intervention ARMA model

If there had been no public heath campaigns on antibiotics consumptions, mean and seasonal function estimated previously would have been similar, thus its were removed to the series on the entire period.
If there had not been public health campaigns on antibiotic consumption, mean and seasonal fuction estimated previously would have been similar. Thus, it was removed to the series during the entire period. To predict what happened during the 2009 A(H1N1) influenza epidemic on outpatient antibiotic use in France, we had to estimate a level change during each campaign period, and we had to predict a future change. The same was done during the opposite period of the year, as changes could also be observed even if campaigns were not running any more. Assuming that a change in the environment at a specific point t causes the mean of the series to change, it is possible to use special types of dummy variables called step functions and impulse functions to build intervention models. Between October 2002 and March 2009, 7 campaigns were run and there were 6 opposite periods. Thus, during the entire study period, 13 intervention functions of the general form $f(\xi_t) = \varpi\xi_t$ were added. The considered inputs were unit step functions and response variables were simple changes in the means (ϖ).

For each campaign, a dummy variable was constructed as follows :

for i=1 to 7, $c_i = \begin{cases} 1 & \text{if week 40 of year } n = (2001+i) \leq \text{t} \leq \text{ week 13 of year } n+1 \\ 0 & \text{otherwise} \end{cases}$

For each rest of period, a dummy variable was constructed as follows :

$$\text{for i=1 to 7, } r_i = \begin{cases} 1 & \text{if week 14 of year } n = (2002+i) \leq \text{t} \leq \text{ week 39 of year } n \\ 0 & \text{otherwise} \end{cases}$$

Assuming that the interventions did not affect the underlying structure of the series, we used the model established for the pre-intervention period to model the entire series.

A general writing of the intervention ARMA model could be:

$$Y_t = \sum_{i=1}^{7} \varpi_i \times c_i + \sum_{i=1}^{6} \omega_i \times r_i + \Theta(B)(\Phi(B))^{-1}\epsilon_t \qquad \epsilon_t \sim N(0, \sigma_\epsilon^2)$$

where $Y_t = X_t - \mu - \mu_w \prod_{i=1}^{7} c_i - \mu_s \prod_{i=1}^{6} r_i - f(t)$ and other notations previously defined.

2.2 Introducing an exogenous variable

To include another time series Z_t, we used models called ARMAX (X for eXogenous) models or "transfer function models". It can help assessing the relations between a target series and one or several explanatory series. Different methodologies have been proposed in the literature to identify the transfer function, pre-whitening both series or one, using times series in their stationary mode ... The transfer function is often quite complex to exploit and interpret. Thus, the simplest one was used; it consisted in a linear regression that we included in the previously identified model (δZ_t).

A general writing of the intervention ARMAX model could be:

$$Y_t = \sum_{i=1}^{7} \varpi_i \times c_i + \sum_{i=1}^{6} \omega_i \times r_i + \delta Z_t + \Theta(B)(\Phi(B))^{-1}\epsilon_t \qquad \epsilon_t \sim N(0, \sigma_\epsilon^2)$$

with previously defined notations.

In the present study, Z_t corresponded to the influenza-like syndrome incidence per 100,000 inhabitants.

2.3 In conclusion

For each considered time series, estimated parameters as well as their standard error are indicated in Table 1. Moreover, p-values of Ljung & Box and Shapiro-Wilk tests are also indicated. A non significant p-value indicates that the null hypothesis was not rejected, corresponding to non correlated residual series and a Gaussian distribution, respectively. Every step previously described was performed using SAS Software version 9.2 (SAS Institute, Inc., Cary, NC), PROC ARIMA with maximum likelihood estimation method.

For each series,

- as expected, winter mean was higher than the summer one, and the seasonal component often included cosines and sines functions of period 26 or 52,
- the underlying ARMA model that generated the series was composed of 2 or 3 multiplicative AR and MA factors modeling short-term, round mid-season and season dependencies (AR process) as well as residual dependencies (MA process),
- most campaign parameters were significant, indicating a decrease compared to the baseline level (June 2000 to October 2002) while other intervention parameters had different profiles between series. For example, for 0-5 years, significant decreases were estimated from 2005, while for >60 years, there was a significant increase since 2004,
- ILS parameter was significant for all the series.

For the entire series, $(X_t)_{t=1,...,455}$, the writing of final model, using previoulsy defined notations, is :

$Y_t = \varpi_1 c_1 + \varpi_2 c_2 + \varpi_3 c_3 + \varpi_4 c_4 + \varpi_5 c_5 + \varpi_6 c_6 + \varpi_7 c_7 + \omega_1 r_1 + \omega_2 r_2 + \omega_3 r_3 + \omega_4 r_4 + \omega_5 r_5 + \omega_6 r_7 +$
$(1 - \theta_7 B^7 - \theta_9 B^9)(1 - \theta_{26} B^{26})((1 - \phi_1 B^1 - \phi_2 B^2 - \phi_4 B^4)(1 - \phi_{30} B^{30})(1 - \phi_{51} B^{51} - \phi_{52} B^{52} - \phi_{53} B^{53}))^{-1} \epsilon_t$
where $Y_t = X_t - (\mu_w \prod_{i=1}^{2} w_i + \mu_s \prod_{i=1}^{3} s_i + \alpha_{c52} \times \cos \frac{2\pi t}{52} + \alpha_{s52} \times \sin \frac{2\pi t}{52})$, $\epsilon_t \sim N(0, \sigma_\epsilon^2)$.

Estimated parameters are indicated in Table S1.

3 Predictions

To make predictions, we first needed to estimate the change (ω_7) between March 2009 and October 2009 (r_7) and the change (ϖ_8) due to the campaign between October 2009 and March 2010 (c_8).
To do so, we used the observed tendency since 2002 and made two scenarios for each series:

-1- **Scenario 1** corresponds to a "stationary" hypothesis: the predicted changes were the same as the ones just previously estimated ($\hat{\omega}_7 = \omega_6$ and $\hat{\varpi}_8 = \varpi_7$),

-2- **Scenario 2** corresponds to an "evolutionary" hypothesis: the mean level changes in antibiotic consumption were predicted: *(i)* using a linear regression on estimated changes since 2002, if there was a clear and continuous tendency since 2002; *(ii)* using a linear regression on estimated changes since a breaking point, if there was a modification in the tendency between 2002 and 2009; *(iii)* using the mean of estimated changes, when oscillations around a value were estimated between 2002 and 2009.

Estimated and predicted changes for both scenarios are presented in Figure S1 for all series.

Forecasts and their 95% confidence intervals (95% CI) were estimated for the two hypotheses. It was first necessary to invert the multiplicative AR factors, because the writing of the ARMA model in a MA(∞) was necessary. This step was realized with Excel 2010 and R2.13.1 Softwares. Forecasts and their 95% CI were validated with SAS for scenario 1 (when estimating intervention ARMA model, SAS used the last estimation of the intervention for predictions). When including a transfer function, if assuming a similar effect during the period of prediction, indicating observed values during this period was sufficient, Z_t for t>455.

Finally, estimated means and trigonometric function were added to the previous forecasts. To caculate the new 95% CI, variance of estimations was added to the one previously calculated, using the well known formula, $Var(aX + bY) = a^2 Var(X) + 2abCov(X, Y) + b^2 Var(Y)$. Stationary step was thus supposed to be independent of the intervention ARMAX models, as well as the centering step with the seasonal component.

For A(H1N1) period considered in the paper, forecasts were summed up for each scenario. 95% CI of those sums were calculated using the sum of the variance of the considered forecasts. A "final" confidence interval was built from these two 95% CI, considering the lowest bound for the lower limit and the highest one for the upper one. We then compared with the observed corresponding sum. Fit of the models and predictions for considered series are presented in Figure S2.

Table S1. Intervention ARMAX model parameter estimates (standard error) and p-value, for each time series.

	Entire series		0-5 years		6-15 years		16-60 years		> 60 years	
μ_w	25.24 (3.29)	-	65.78 (13.75)	-	26.68 (7.04)	-	21.23 (2.52)	-	19.16 (3.07)	-
μ_s	16.54 (4.16)	-	38.40 (14.60)	-	16.04 (5.32)	-	17.70 (3.40)	-	13.60 (2.50)	-
α_{c52}	-1.15 (.457)	.01	-	-	-	p	-1.04 (.376)	.007	-0.65 (.319)	.04
α_{s52}	-1.06 (.453)	.02	-	-	-3.51 (.726)	<.0001	-0.982 (.373)	.001	-1.69 (.317)	<.0001
α_{c26}	-	-	-	-	-	-			-	-
α_{s26}	-1.36 (.456)	.004	-8.67 (1.67)	<.0004	-	-			-	-
AR F1†										
ϕ_1	0.49 (.048)	<.0001	0.73 (.043)	<.0001	0.93 (.043)	<.0001	0.43 (.047)	<.0001	0.33 (.047)	<.0001
ϕ_2	0.11 (.049)	.021	-0.12 (.043)	.005	-0.29 (.043)	<.0001	0.14 (.048)	.003	0.20 (.047)	<.0001
ϕ_4	-0.16 (.043)	.0002	-	-	-	-	-0.20 (.043)	<.00001	-	-
ϕ_6	-	-	-0.14 (.039)	.0002	-	-	-	-	-	-
AR F2†										
ϕ_{11}	-	-	-	-	-	-	-	-	-0.12 (.047)	.01
ϕ_{17}	-	-	-	-	-	-	-	-	-0.13 (.048)	.005
ϕ_{25}	-	-	0.14 (.045)	.002	-	-	-	-	-	-
ϕ_{30}	-0.12 (.048)	0.014	-	-	-	-	-0.14 (.049)	.004	-	-
AR F3†										
ϕ_{51}	0.13 (.033)	.0001	-	-	-	-	0.14 (.036)	<.0001	0.12 (.037)	.001
ϕ_{52}	0.64 (.038)	<.0001	.98 (.005)	<.0001	0.97 (.008)	<.0001	0.56 (.040)	<.0001	0.59 (.040)	<.0001
ϕ_{53}	0.13 (.033)	<.0001	-	-	-	-	0.19 (.036)	<.0001	0.16 (.37)	<.0001
MA F1†										
θ_5	-	-	-	-	-	-	-	-	0.11 (.047)	.02
θ_7	-0.13 (.049)	.007	-0.21 (.052)	<.0001	-0.16 (.045)	.0004	-0.12 (0.049)	0.02	-	-
θ_9	-0.10 (.047)	.044	-0.12 (.044)	.008	-0.12 (.044)	.008	-	-	-	-
MA F2†										
θ_{26}	0.20 (.046)	<.0001	-	-	-	-	0.21 (.017)	<.0001	-	-
θ_{51}	-	-	-0.10 (.032)	.001	-	-	-	-	-	-
θ_{52}	-	-	0.65 (.050)	<.0001	0.68 (.055)	<.0001	-	-	-	-
θ_{54}	-	-	-	-	-	-	-	-	0.15 (.052)	.003
σ^2_ϵ	1.829	-	14.256	-	2.786	-	1.345	-	1.516	-
ϖ_1	-2.54 (.506)	<.0001	0.075 (1.54)	.96	-2.51 (.870)	.004	-3.01 (.372)	<.0001	-1.44 (.375)	.0001
ϖ_2	-3.43 (.661)	<.0001	-1.00 (1.73)	.56	-4.38 (.936)	<.0001	-3.38 (.486)	<.0001	-0.98 (.460)	.03
ϖ_3	-4.75 (.751)	<.0001	-7.92 (1.86)	<.0001	-6.26 (.970)	<.0001	-3.97 (.550)	<.0001	-0.63 (.512)	.22
ϖ_4	-6.14 (.799)	<.0001	-16.19 (1.98)	<.0001	-5.18 (1.01)	<.0001	-4.88 (.581)	<.0001	-1.88 (.539)	.0005
ϖ_5	-7.31 (.832)	<.0001	-19.03 (2.08)	<.0001	-8.01 (1.04)	<.0001	-5.56 (.605)	<.0001	-2.32 (.558)	<.0001
ϖ_6	-6.65 (.863)	<.0001	-19.07 (2.17)	<.0001	-8.08 (1.06)	<.0001	-4.58 (.631)	<.0001	-0.47 (.582)	.42
ϖ_7	-6.28 (.902)	<.0001	-18.05 (2.30)	<.0001	-7.78 (1.14)	<.0001	-4.27 (.665)	<.0001	0.21 (.598)	.73
ω_1	-0.50 (.503)	.32	-1.30 (1.56)	.40	-2.95 (.859)	.0006	-0.40 (.376)	.29	-0.06 (.371)	.88
ω_2	-0.07 (.647)	.91	-3.22 (1.71)	.06	-3.597 (.911)	<.0001	-0.07 (.480)	.88	0.49 (.453)	.28
ω_3	-0.48 (.728)	.51	-9.95 (1.84)	<.0001	-3.40 (.945)	.0003	-0.16 (.539)	.76	1.03 (.502)	.04
ω_4	-0.93 (.781)	.24	-13.52 (1.96)	<.0001	-5.63 (.985)	<.0001	-0.24 (.575)	.68	1.03 (.534)	.05
ω_5	0.22 (.820)	.79	-11.95 (2.06)	<.0001	-5.04 (1.01)	<.0001	0.79 (.604)	.19	2.42 (.556)	<.0001
ω_6	0.31 (.862)	.71	-13.84 (2.16)	<.0001	-5.44 (1.04)	<.0001	0.78 (.639)	.22	2.61 (.581)	<.0001
δ	0.007 (.0006)	<.0001	0.019 (.002)	<.0001	0.01 (.001)	<.0001	.005 (.0005)	<.0001	.005 (.0006)	<.0001
L-B‡	-	.51	-	.86	-	.79	-	.69	-	.97
S-W††	-	.11	-	.10	-	.23	-	.08	-	.10

†: multiplicative AR or MA factor.

‡: Ljung & Box test.

††: Shapiro-Wilk test. To not rejected null hypothesis, between 2% and 5% of residuals were removed.
